# Non-Destructive Detection of Abnormal Chicken Eggs by Using an Optimized Spectral Analysis System

**DOI:** 10.3390/s22249826

**Published:** 2022-12-14

**Authors:** Juntae Kim, Dennis Semyalo, Tae-Gyun Rho, Hyungjin Bae, Byoung-Kwan Cho

**Affiliations:** 1 Department of Biosystems Machinery Engineering, College of Agricultural and Life Science, Chungnam National University, 99 Daehak-ro, Yuseoung-gu, Daejeon 34134, Republic of Korea; 2 Department of Smart Agricultural Systems, College of Agricultural and Life Science, Chungnam National University, 99 Daehak-ro, Yuseoung-gu, Daejeon 34134, Republic of Korea

**Keywords:** spectral analysis, nondestructive measurement, abnormal egg detection, waveband selection, system optimization

## Abstract

Environmental pressures, such as temperature and light intensity, food, and genetic factors, can cause chicken eggs to develop abnormalities. The common types of internal egg abnormalities include bloody and damaged egg yolk. Spectrometers have been recently used in real-time abnormal egg detection research. However, there are very few studies on the optimization of measurement systems. This study aimed to establish optimum parameters for detecting of internal egg abnormalities (bloody and damaged-yolk eggs) using visible and near-infrared (Vis/NIR) spectrometry (192–1110 nm range) and multivariate data analysis. The detection performance using various system parameters, such as the types of light sources, the configuration of the light, and sensor positions, was investigated. With the help of collected data, a partial least-squares discriminant analysis (PLS-DA) model was developed to classify normal and abnormal eggs. The highest classification accuracy for the various system parameters was 98.7%. Three band selection methods, such as weighted regression coefficient (WRC), sequential feature selection (SFS), and successive projection algorithm (SPA) were used for further model optimization, to reduce the spectral bands from 1028 to less than 7. In conclusion the results indicate that the types of light sources and the configuration design of the sensor and illumination affect the detection accuracy for abnormal eggs.

## 1. Introduction

The nutritional value of eggs increases global egg consumption. As egg consumption increases, the demand for safe, hygienic, and high-quality eggs also increases. This condition necessitates a screening technology for pre-sorting and removal of abnormal eggs, which is becoming increasingly important [1]. In recent years, the incidence of abnormal eggs has decreased due to the development of pure line selection and cross-breeding techniques, aviary lighting control programs, and feed improvements [2]; however, the abnormal condition is still prevalent. If abnormal eggs enter the commercial market or are sold, they induce aversion and have a detrimental impact the poultry industry and food hygiene, causing bacterial infection and food spoilage; therefore, they must be minimized. Abnormal egg types include those with blood spots, meat spots, bloody eggs, putrefactive eggs, yolk-destroyed eggs, yolkless eggs, speckled eggs, pimpled eggs, eggs with calcium deposits, and misshaped eggs [3]. Among all, bloody eggs and meat spots were the most common types of internally abnormal eggs. Bloody eggs are eggs with mixed blood inside and meat spots in an eggshell pigment or oxidized blood vessel [4]. Although it depends on the amount of blood, eggs with blood spots and meat spots are also commonly referred to as bloody eggs. In the case of blood spots, the capillary vessel bursts into the egg yolk when it is formed. It usually appears on the egg yolk surface. Severe bloody eggs are rare; however, the entire egg albumen is congealed and pink [5]. There are various causes of bloody eggs, but it is known that the breed of laying hens, poor feeding, intermittent and continuous lighting, age of the chickens, and temperature of the cage can have an adverse effect [5,6]. In addition, yolk-destroyed eggs are caused by external influences such as the centrifugal force when the eggs are rolled during the laying process. Further, the decayed/rotten eggs may occur when external microbes enter the egg due to eggshell damage or when eggs stay in the corner of a cage for a long-time during summer, resulting in spoilage. Egg industries use egg sorters for rapid and non-destructive detection of problematic internal abnormalities. 

Many researchers have conducted studies on non-destructive sorting techniques to classify eggs. Most screening techniques for detecting bloody eggs include spectrometer-based detection methods and Red Green Blue (RGB) and hyperspectral image analysis [7,8,9,10]. Chen et al. [9] demonstrated a visible near-infrared (Vis/NIR) spectrometer in the range of 200–1100 nm on a roller conveyer belt that passed four eggs per second and detected the artificially prepared bloody eggs by injecting 0.05 mL of chicken blood into brown eggs. It has been reported that bloody eggs were classified with an accuracy of 96.9% using the binary logistic regression technique. Omid et al. [7] classified internally abnormal white eggs by analyzing RGB images obtained using a light-projection-type screening machine. It was reported that bloody eggs were classified with 98% accuracy using a fuzzy-classification model. Feng et al. [10] attempted to classify bloody eggs using a hyperspectral imaging device in the 450–1000 nm wavelength region. The obtained spectral images were analyzed using a support vector machine, which showed that classification was possible with an accuracy of 96.4%. However, to date, methods for detecting bloody eggs through image analysis have been limited to the sorting of white eggs. Hyperspectral imaging systems have not been easily applied to real-time online detection of abnormal eggs because of their high cost and slow scanning speed. Therefore, most currently used commercialized systems for egg quality measurement adopt spectrometer-based technology. Although several studies have been conducted on detecting internal abnormal eggs using a spectrometer, studies related to optimization for system parameters, such as the types of light sources, the configuration of the light, and sensor positions for detecting abnormal eggs have not been attempted. 

In reality, detecting bloody eggs using a spectrometer is more complex for brown than for white eggs. This is due to protoporphyrin IX (PPIX), a pigment found in brown eggs [11,12]. PPIX is also present in white-shell eggs and blue-shell eggs, but its content is low [13]; therefore, it does not pose a significant problem while detecting bloody eggs. Improved breeding techniques also produce white eggshells with high PPIX content; however, it is still relatively easier to detect bloody eggs among them than brown eggs [1]. Various studies have been conducted on bloody egg detection [7,8,9,10,11,12,14]; however, no specific research is available on optimizing of the system parameters, such as wavebands, light source, and position of components, for abnormal egg detection. This study was conducted under the hypothesis that optimization of the spectral system parameters would increase the abnormal egg detection performance. The objective of this study was to investigate the optimal condition of the system parameters, such as the type of light source, the configuration of illumination and detector, and the waveband combination for the detection of internal abnormal eggs, such as bloody eggs, and yolk-destroyed eggs.

## 2. Materials and Methods

### 2.1. Sample Preparation

The regular eggs and bloody eggs within 24 h of spawning used in this experiment were obtained from a local grading and packaging center in Anseong, Korea. Upon returning to the laboratory from the farm, all egg samples were checked using the light transmission method, and cracked eggs were removed. 

**Figure 1:**
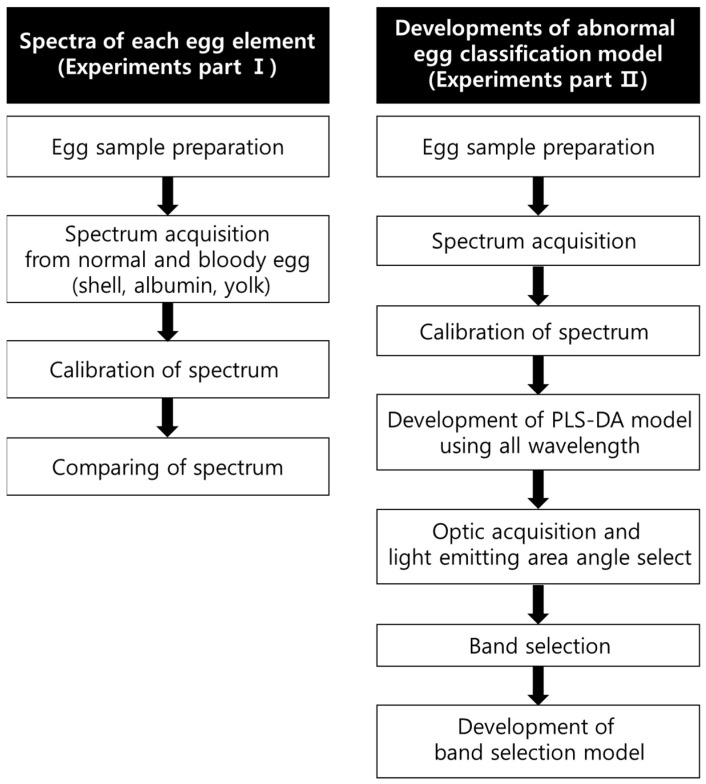
Experiment flow chart for the development of the abnormal egg detection algorithm.

The overall experimental flow is as the Figure 1. The difference in wavelength for each component of internally abnormal eggs and normal eggs was investigated using gold- and silver-coated lamps. Silver-coated lamps and gold-coated lamps are generally used in spectrometer-based sorters. The selection of a specific light source increases the sorting performance efficiency.

Ten normal, five bloody, five meat spot, and five blood spot egg samples were used to check the difference in wavelength for each component of bloody and regular eggs using gold and silver-coated lamps. The normal and bloody egg samples were broken up, divided into albumen, yolk, meat and blood spots, and eggshells, respectively, and stored in a Petri dish (diameter, Ø: 90 mm; height: 15 mm). The eggs were stored at 5 °C before the measurement. 

**Figure 2:**
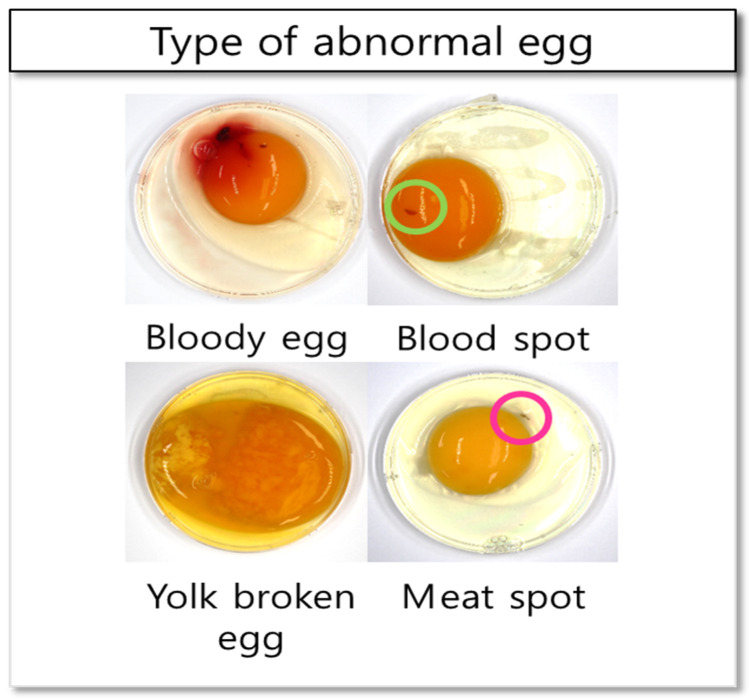
Sample images of yolk-destroyed eggs and bloody eggs.

After investigating the spectral characteristics of each component of the normal and bloody egg, 50 normal eggs, 50 bloody eggs, and 50 yolk-destroyed eggs (Figure 2) were measured to build an initial classification model and optimize the waveband combination for abnormal egg sorting according to the position of illumination and the sensor. Egg shakers (SY-A001, FTVOGUE, China) were used to produce yolk-destroyed egg samples by rotating the normal eggs for 1 min. All the egg samples (normal eggs, bloody eggs, and yolk-destroyed eggs) were stored at 5 °C before the experiment.

### 2.2. Hardware Setup

**Figure 3:**
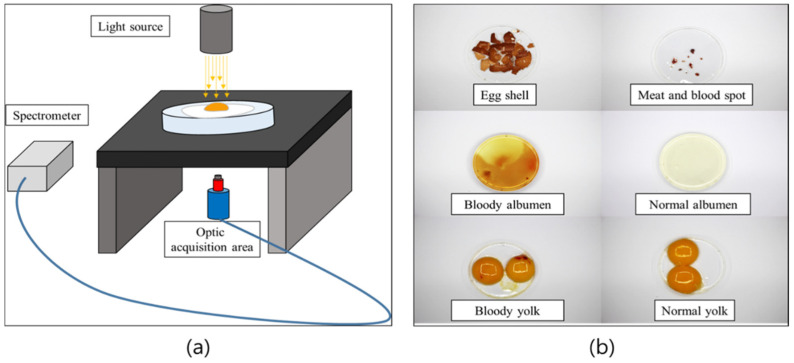
Spectrum acquisition system setup for determining each egg element: (**a**) schematic of the acquisition system; (**b**) sample of each egg element.

Spectral measurements were performed using a spectrometer (Ocean HDX, Ocean Insight, Inc., New York, NY, USA) capable of detecting wavelengths (192–1110 nm) in the Vis/NIR region using Ocean View 2.0.8 software (version 2.0.8, Ocean Insight, Inc., New York, NY, USA). The spectral resolution of the spectrometer was 0.17 nm, consisting of a total of 2068 wavelengths. A sample stage for spectral measurements (Figure 3a,b) of each egg element (eggshell, meat spot or blood spot, bloody albumen, bloody yolk, normal albumen, and normal yolk) was prepared. A focusing lens was installed in the light-emitting part so that the light source could illuminate only the region of interest. The light-receiving part was placed under the sample stage, and a disk with a field of view (FOV) of 1° was installed in front of the Gershun tube (Ocean Insight, Inc., New York, NY, USA) to reduce the amount of incoming light and prevent light saturation. For data acquisition, gold-coated (JCR12V, 100 W; Ushio Inc., Yokohama-shi, Japan) and silver-coated halogen lamps (JCR12V, 100 W; Ushio Inc., Japan) were used for sample illumination.

### 2.3. Spectral Acquisition

**Figure 4:**
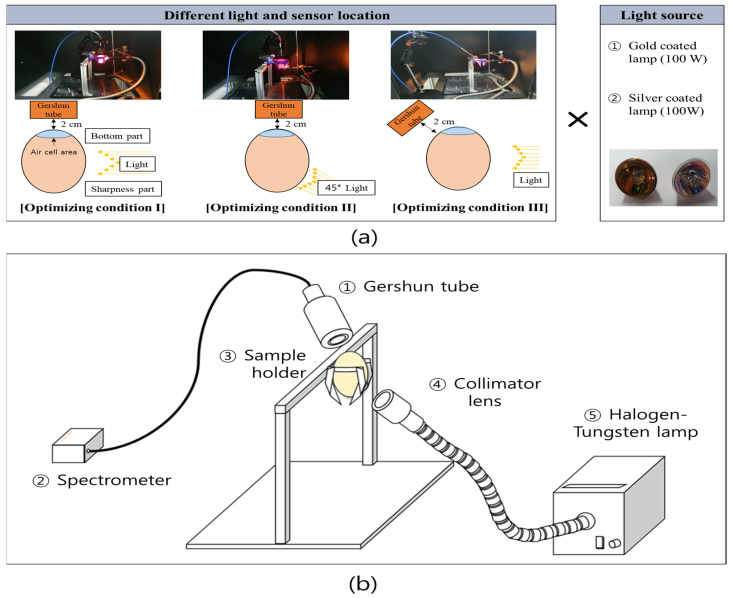
Experimental design schematic. (**a**) Each experimental condition schematic; (**b**) abnormal egg measurement system schematic.

After the spectral acquisition of each normal and bloody egg element, an initial predictive model was developed for the detection of abnormal eggs. The model for classifying abnormal eggs was constructed using a 3 × 2 factorial design method. Six groups were set up using three different setting conditions of light-receiving and light-emitting parts, where light saturation did not occur, and halogen lamps were coated with two kinds of metals (gold and silver). A Gershun tube was mounted on the light-receiving part and on a disk with a FOV of 10° in front of the Gershun tube to increase the light signal of the egg sample (Figure 4). When measuring the spectrum of the egg, the blunt end of the egg where the air cell was located, faced upward for the spectral measurement. The measurement condition of the spectrum was set to a 10 ms integration time, and the spectra of 10 measurements for each sample were averaged and used for analysis. Two types of light sources, gold-coated and silver-coated halogen-tungsten lamps, were installed in the light bank (LS-F100HS) and used to illuminate the eggs through a light guide (GS5-100F, Seokwang Optical Co., Ltd., Hwaseong-shi, Korea). A collimator lens (AL-15H, Seokwang Optical Co., Ltd., Korea) was used to collect light with a diameter of 10 mm at a working distance (W/D) of 20 mm so that light could illuminate only a certain area of the egg. The light-receiving part (the light fiber connected to the spectrometer), which acquires light transmitted from the egg, was positioned at 10 mm from the egg. The light-receiving and light-emitting parts were constructed with three different configurations (I–III) to determine the optimized measurement conditions, as shown in Figure 4a,b. In all cases, the light-emitting part was placed 20 mm away from the egg. The spectra of 150 eggs (50 eggs, 50 normal eggs, and 50 yolk-destroyed eggs) were measured five times by rotating each egg, and 750 spectra were obtained.

### 2.4. Intensity Calibration

The intensity calibration was performed to convert the raw intensity value to the relative transmittance value. The intensities of the white reference with a Teflon disk and the dark reference in the darkroom state were measured for intensity calibration. The calculation for the calibration was done by following Equation (1), where ‘*W*’ is the transmittance intensity value of the white reference, and ‘*D*’ is the intensity value of the dark room condition. ‘*B*’ is the intensity of the acquired transmittance spectrum value for each wavelength [15]:

(1) $$\mathrm{Calibration}\;\mathrm{sample}\;\mathrm{band}=\frac{B-D}{W-D}$$

For the dark reference measurement used for calibration, the spectrum was measured 10 times by closing the cover of the Gershun tube in a dark room. For the measurement of the white reference, a white Teflon disk with a thickness of 0.5 mm and a diameter (Ø) of 10 mm was mounted in front of the Gershun tube without an egg and measured 10 times. Subsequently, the average value of the obtained spectra was used for the calibration.

### 2.5. Data Preprocessing

The acquired spectrum includes various types of noise such as random noise, variation from different sample shapes and the direction of light, and light scattering generated from the sample. Therefore, applying a spectral preprocessing algorithm is necessary to minimize unnecessary noise in the data [16]. Spectral preprocessing methods, including normalization methods (minimum, maximum, and range normalization) [17], standard normal variate (SNV) [18], multiplicative scatter correction (MSC) [19], and Savitzky-Golay (SG) 1st and 2nd derivative for spectral pretreatment [20] were used in this study. In general, SNV and MSC normalization methods reduce sample-to-sample variability and adjust the baseline shift between samples. The SG 1st derivation removes the baseline of the spectrum, and the SG 2nd derivation removes the baseline and linear trend of the spectrum [16].

### 2.6. Model Development

The partial least-squares discriminant analysis (PLS-DA) method was used to develop a classification model to predict three different egg groups (normal eggs, bloody eggs, and yolk-destroyed eggs). Each group reference value was labeled as a number for PLS-DA analysis: the normal egg was labeled as ‘0’ (dependent variable Y = 0), the bloody egg spectrum data as ‘1’ (dependent variable Y = 1), and the yolk-destroyed egg group as ‘2’ (dependent variable Y = 2).

(2) $$Y=\{\begin{array}{l} \;0=\mathrm{Normal}\;\mathrm{egg}\;\mathrm{group}\\ \;1=\mathrm{Bloody}\;\mathrm{egg}\;\mathrm{group}\\ \;2=\mathrm{Yolk}-\mathrm{destroy}\;\mathrm{egg}\;\mathrm{group} \end{array}$$

PLS algorithm is as the following equation:

(3) $$X={\mathrm{TP}}^{T}+E$$

(4) $$Y={\mathrm{UQ}}^{T}+F$$

Y is a matrix of dependent variables containing reference values that define the condition of the egg (0, 1, and 2). X is an n × p matrix of independent variables corresponding to each spectral variable. n is the number of sample wavebands and p is the number of wavebands. Matrix X is decomposed into the loading matrix P, score matrix T, and error matrix E. Matrix Y is composed of a loading matrix Q, score matrix U, and error matrix F. For the development of the classification model, 70% of the 750 spectral data points were randomly assigned to the calibration set, and the remaining 30% were assigned to the validation set.

### 2.7. Waveband Selection Methods

Because most egg grading lines in Korea measure 10 eggs per second, the per-egg-spectra analysis time is a critical consideration. Herein, extraction of a few influential wavebands from the multitude acquired from the spectrometer can reduce the processing time per egg. In this study, four band selection methods, weighted regression coefficient [21], sequential feature selection [22], successive projection algorithm [23], and stepwise regression [24] were used to investigate the important wavelengths for discriminating abnormal eggs.

#### 2.7.1. Weighted Regression Coefficient (WRC)

By measuring the weight, the weighted regression coefficient (weighted β-coefficient) calculates the correlation between the wavelength of each predictor variable and the corresponding response variable. In the WRC, auto-scaling was performed on the same scale by dividing each spectral wavelength by the standard deviation of the PLS-DA model. Generally, a higher magnitude of the beta coefficient indicates a variable that contributes more to building the model. Therefore, wavelength regions with higher beta coefficients are commonly selected as the important variables. However, the transmittance value of the original sample spectrum must be considered when selecting the primary wavelength. For example, a variable with a high beta coefficient and low spectral intensity in the waveband may contribute less to the model prediction.

#### 2.7.2. Sequential Feature Selection (SFS)

Sequential feature selection (SFS) is a method that repeatedly selects features individually by checking the optimal model fit. The SFS consists of a criterion function and a sequential search algorithm. The algorithm starts with an empty set and sequentially adds features with high weights to the model based on a criterion function [22]. The reference function can be defined in two ways. In the case of a classification model, the reference function is the misclassification rate, and in the case of a regression model, it is the mean square error. The search algorithm continued the same process until the required number of variables was selected.

#### 2.7.3. Successive Projection Algorithm (SPA)

A successive projection algorithm (SPA) is used to reduce collinearity between variables. The SPA is used to select the optimal variable by minimizing the overlapping information included in the spectral information. The SPA algorithm starts with a single waveband or variable, adds a new variable with the maximum projection and repeats the process until the desired number of variables is selected. Thus, the algorithm produces a subset of variables with the least linear relationship between them from all the variables of the training set and applies this set to the cross-validation dataset to evaluate performance [25,26]. SPA is widely used to select the optimal number of variables in multivariate quantitative and qualitative analyses.

#### 2.7.4. Stepwise Regression (SR)

The SR selection method is the most straightforward and practical method for the variable selection of multiple linear regression models. A SR model is a method of adding or removing variables based on the statistical significance in a regression model. In this method, the *p*-value for the F-statistic is calculated to test the model with or without a potential variable. Stepwise selection methods are divided into forward and backward selection methods. This study uses the forward selection method to add variables to the model. In the case of the spectrometer used in this study, the spectral resolution is 0.17 nm, so each wavelength is very close. Therefore, in the case of a wavelength selected by the WRC, SFS, or SPA methods, there is a possibility that the repeat may choose a wavelength in a close and similar region, and there is also a possibility that it may overlap with the wavelength information. However, this method is not effective in reducing the number of bands. Therefore, SR analysis was applied in this study to remove the overlapping spectral regions. The model was applied to select 10 wavebands from the spectra obtained with silver and gold lamps using the WRC, SFS, and SPA waveband selection methods. The analysis was conducted by setting the threshold for the maximum *p*-value of the waveband to 0.05, 0.01, 0.001, and 0.0001.

### 2.8. Model Performance Assessment

The accuracy obtained from the training and test sets for the developed model was evaluated, and the beta coefficient was obtained according to the preprocessing method. The formula used to evaluate accuracy is as follows:

(5) $$\mathrm{Accuracy}\left(\%\right)=\frac{\mathrm{Tp}+\mathrm{Tn}}{\mathrm{Tp}+\mathrm{Tn}+\mathrm{Fp}+\mathrm{Fn}}\times 100$$

In Equation, ‘Tp’ means true positive (the number of normal eggs detected as normal eggs), ‘Fn’ means false negative (the number of normal eggs detected as abnormal eggs), ‘Tn’ means true negative (the number of abnormal eggs detected as abnormal eggs), and ‘Fp’ means false positive (the number of abnormal eggs detected as normal eggs). All wavelength analyses were performed using MATLAB, version 2021b (MathWorks, Natick, MA, USA).

## 3. Results and Discussion

### 3.1. Illumination Optimization

**Figure 5:**
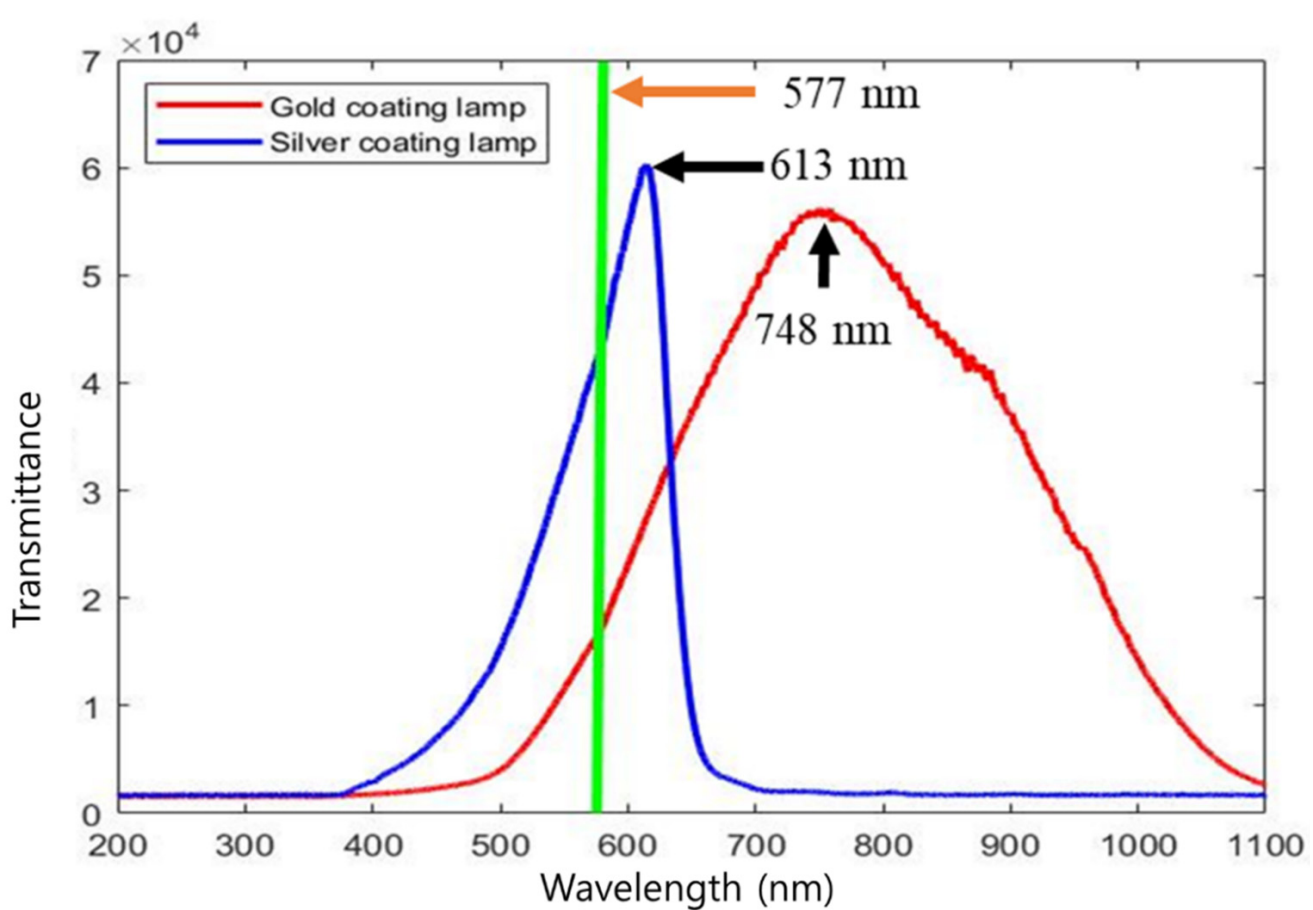
The light patterns of the gold-coated halogen-tungsten lamp and the silver-coated halogen-tungsten lamp.

The spectral characteristics of the gold- and silver-coated halogen lamps used in this study are shown in Figure 5. The silver-coated lamp emitted light in the wavelength range of 400–700 nm, and the gold-coated lamp emitted light in the range of 500–1100 nm. These phenomena are caused by the differences in the extinction coefficient and refractive index depending on the coated mineral. The silver-coated halogen lamp showed a higher intensity in the 565–585 nm range, known as the blood-sensitive region, than the gold-coated halogen lamp. The light intensity in the 577 nm region, where hemoglobin reacts, was also higher than that of the gold-coated lamp. The molecular structure of PPIX is similar to hemoglobin (Hb), and the spectral absorbance regions are identical. The absorption wavelengths of PPIX were 539, 589, and 643 nm [14], and those of hemoglobin were 415, 539, and 577 nm, respectively [27]. Eggshells are composed of calcium carbonate (CaCO_3_) crystals. The calcium carbonate ions (CO_3_^2−^) absorb light with a wavelength of less than 250 nm; therefore, the transmittance of light below 500 nm in eggs is generally low [1,27]. For this reason, the wavelength 415 nm cannot be used for bloody egg detection, and the wavelength 539 nm overlaps with the PPIX wavelength, preventing the acquisition of spectral information. Therefore, bloody egg separation can be accomplished using a wavelength of 577 nm. However, this may interfere with detection because of the PPIX detection wavelength of 589 nm [28]. Hence, the detector’s accuracy varies depending on how quickly and accurately the wavelength of 577 nm can be used to detect bloody eggs.

The spectrometer did not detect light over 700 nm when a silver-coated lamp was used. Therefore, the difference in the light intensity range for each light source can reduce noise and information in the unnecessary range and increase hemoglobin classification accuracy. Therefore, in this research, to reduce the noise and number of wavelengths of the classification model, only a wavelength range from 500 to 680 nm was selected from the entire wavelength range (192–1110 nm).

### 3.2. Egg Component Spectra

**Figure 6:**
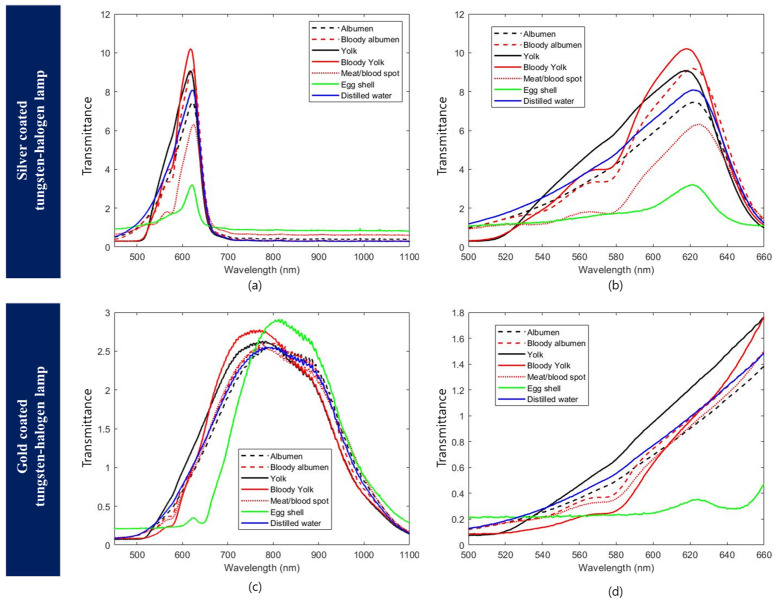
Comparison of gold and silver light patterns for normal and abnormal egg elements: (**a**,**b**) The spectrum for each element of abnormal and normal eggs obtained through a silver-coated halogen-tungsten lamp; (**c**,**d**) The spectrum for each element of abnormal and normal eggs obtained through a gold-coated halogen-tungsten lamp.

Figure 6 shows the results of checking the spectra of the silver-coated lamp and gold-coated lamp for each element by separating the eggs into each component. When examining the wavelengths obtained with the silver- and gold-coated lamp in transmittance mode for bloody egg white, bloody egg yolk, and blood spot/meat spot, the intensity of the components in the 565–585 nm region was lower than that of normal eggs (Figure 6b,d). This is because hemoglobin absorbs light in response to wavelengths in the range of 577 nm. Brant et al. [14] measured the spectrum of white-shell eggs mixed with blood using the light transmittance method. It was reported that bloody albumen and albumen containing meat/blood spots had a lower wavelength intensity than normal egg whites. Eggshells are composed of calcium carbonate, and it is known that calcium carbonate blocks wavelengths below 550 nm. This study also confirmed that the wavelength intensity increased above 550 nm when the silver and gold lamps were used. The eggshell contained PPIX, which is known to absorb light at 643 nm [14]. Similarly, a decrease in wavelength intensity was also observed in the 643 nm region in this study.

### 3.3. Abnormal Egg Detection Model

#### 3.3.1. Raw Data Spectra

**Figure 7:**
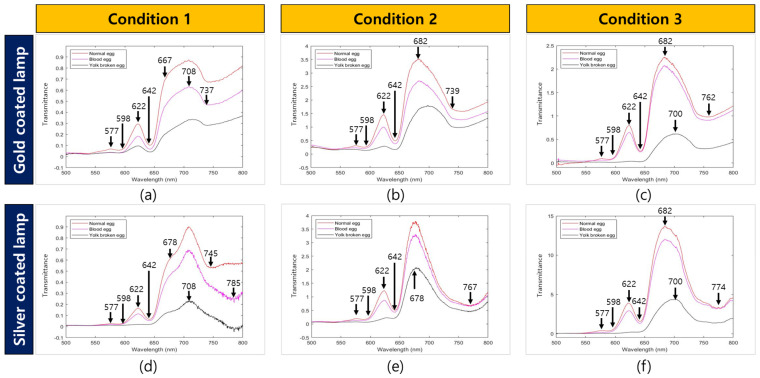
Abnormal egg and normal egg mean spectrum according to various light test conditions. (**a**) Gold−coated condition 1 mean spectrum; (**b**) gold−coated condition 2 mean spectrum; (**c**) gold−coated condition 3 mean spectrum; (**d**) silver−coated condition 1 mean spectrum; (**e**) silver−coated condition 2 mean spectrum; (**f**) silver−coated condition 3 mean spectrum.

Figure 7 shows the average spectrum of the different angles of the incident light and the transmitted light for each type of lamp. The results of the average spectra indicated that the spectral intensity of bloody eggs and yolk-destroyed eggs was lower than that of normal eggs. Titova et al. [29] reported that the spectral intensity of decayed and bloody eggs was lower than that of normal eggs in an experiment with Vis/NIR transmittance measurements in 550–850 nm. Furthermore, Brant et al. [14] reported a reduced spectral intensity of bloody eggs compared to normal eggs. Chen et al. [9] reported a difference in the wavelength between normal and bloody eggs in the 500–600 nm region using the Vis/NIR absorption spectral measurement. The spectral intensity of bloody eggs increases in the absorbance method because hemoglobin absorbs light, and vice versa in the transmittance method [9,14,29]. The spectral shape of the bloody eggs obtained in this experiment was the same as that reported previously.

In the case of the gold-coated lamp under condition 1, no spectral difference was observed between the bloody egg sample and the yolk-destroyed egg sample in the 550–600 nm region, unlike the silver-coated lamp spectrum result of condition 1. By comparing the wavelength changes under conditions 1 and 2, it was possible to confirm the difference in the spectral pattern. In the condition 1, an increased intensity was confirmed in the range of 642–708 nm. However, in the condition 2, the spectral intensity increased before the range of 642–682 nm and then decreased.

#### 3.3.2. Model Based on All Wavebands

**Figure 8:**
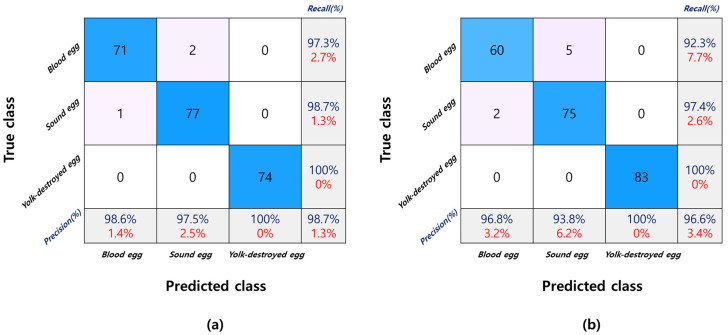
Confusion matrix of condition two gold−coated and silver−coated lamp model. (**a**) Gold-coated halogen tungsten lamp model; (**b**) Silver−coated halogen tungsten lamp model. The values in the parenthesis are errors.

Table 1 shows the results of the abnormal egg classification model according to the type of light source (silver coating vs. gold coating) and the angle of the light-emitting unit and light-receiving unit (conditions 1–3). It shows that the classification accuracy of blood and yolk-destroyed eggs were low (78.4–85.2%) with gold-coated lamps under condition 1. The classification accuracy of the gold-coated lamp model with condition 1 is inferior because the wavelength region of the yolk-destroyed eggs overlapped with the wavelength region of the bloody eggs. When comparing the classification accuracy of each condition, condition 2 showed a relatively high classification accuracy for both the gold- and silver-coated lamps (92.0–98.7%), and the gold-coated lamp with mean normalization preprocessing showed the highest accuracy. Chen et al. [9] classified sound and blood spot eggs using a spectrometer in the VIS/NIR region and showed an accuracy of 90.6% when using PLS-DA, and 96.9% for BLR accuracy was reported. The results of this study showed high numerical accuracy than this research. Figure 8a,b show the confusion matrix of condition 2 for the gold-coated and silver-coated models. The classification model result of egg yolk destruction was 100% for both types of lamps. However, the gold-coated lamp model showed a high prediction rate in overall accuracy (%), and the gold-coated lamp model showed higher classification accuracy even when comparing recall and precision results than the silver-coated lamp model. Lee et al. [8] conducted a blood egg-sorting experiment using Vis/NIR spectrometer. Additionally, he reported that the PLS-DA model accuracy was more than 95%, which is suitable for the sorting machine. Condition 2 also showed more than 95% sorting accuracy in this study. The result indicates that the proposed method can be used for the actual egg sorting machine. In the case of the model precision for the sound egg (93.8%) measured with silver coated lamp was lower than that for the egg with the gold-coated lamp (97.5%). The model precision of the blood egg was also higher that for the egg with the gold-coated lamp. However, the yolk-destroyed egg precision of gold-coated and silver-coated lamp models showed 100%. In the case of recall (%), also known as sensitivity (%), the gold-coated lamp model showed higher values in every group (blood egg, sound egg, yolk-destroyed egg).

**Figure 9:**
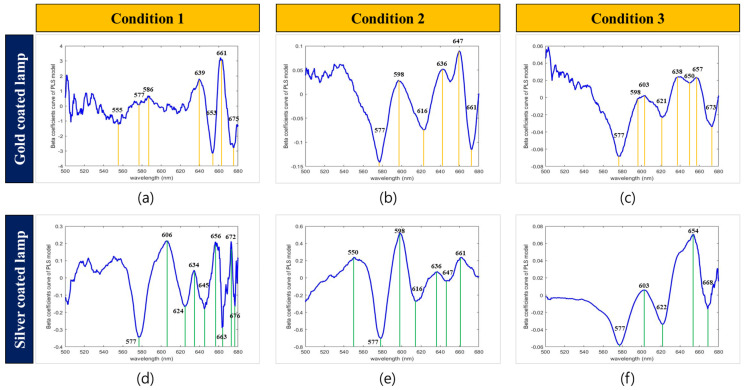
The beta coefficient value of the abnormal egg classification model. (**a**) Gold−coated condition 1 beta coefficient; (**b**) gold−coated condition 2 beta coefficient; (**c**) gold−coated condition 3 beta coefficient; (**d**) silver−coated condition 1 beta coefficient; (**e**) silver−coated condition 2 beta coefficient; (**f**) silver-coated condition 3 beta coefficient.

The beta coefficient showed the maximum absolute value in the wavelength region of 577 nm under all conditions except for condition 1. In general, the beta coefficients with the largest magnitudes are the most influential on the developed model. Therefore, in the model constructed in this experiment, the absorption wavelength region of hemoglobin (577 nm) had the most significant influence on model construction. As a result of confirming the beta coefficient in Figure 9a, it was confirmed that the weight of the 640–680 nm region was important when developing the model. The range of 640–680 nm is the red visible light region, which is the weight for eggshell color. At 600–620 nm, the beta coefficient decreased rapidly, caused by the difference in the spectral intensity of normal eggs and yolk-destroyed eggs in the area.

The gold-coated lamp model under condition 2 showed a high beta coefficient weight in the 539 nm region, which is the spectral region of PPIX, and a high beta coefficient value in the 647 nm region near 643 nm. In general, wavelengths of 539, 589, and 643 nm are known as the absorption wavelengths of PPIX, and wavelengths of 415, 539, and 577 nm are known as hemoglobin-derived wavelengths [14,26]. It was confirmed that PPIX and hemoglobin components affected model development. The silver-coated lamp model under condition 2 confirmed the main wavebands at 577 and 598 nm. The 577 nm waveband is the hemoglobin region and 598 nm is not a specific chemical component wavelength. Nevertheless, the difference in the band ratio between 577 and 598 nm can help in bloody egg classification. Rahman et al. [26] used a band ratio of 575/598 nm to detect the sex of early stage chicken eggs and reported a higher rate of hemoglobin production in males than in females. For this reason, a decrease in transmittance intensity due to absorption at a wavelength of 575 nm, which is related to hemoglobin, was observed. In this study, the beta coefficient of every model, except for the gold-coated lamp of condition 1, showed high beta coefficients at wavelengths of approximately 577 nm and 598 nm. In this study, condition 2 (Figure 4a) showed the highest abnormal egg classification accuracy with gold and silver coatings. This suggests that the light transmission position of the egg varies depending on the angle of the light-emitting unit and light-receiving unit, and the classification accuracy may vary depending on the angle of the light-emitting unit and light-receiving unit. Therefore, it is crucial to set an optimal angle to detect small blood and meat spots.

#### 3.3.3. Model Based on Selected Wavebands

Band selection was selectively conducted on the data obtained under condition 2, which showed the best model classification accuracy. As for the spectral region, the 550–600 nm region showed the highest weight based on the beta coefficient of condition 2; therefore, this region was selected to remove band noise generated by other materials. We used three variable selection methods (WRC, SFS, and SPA) and SR for wavelengths selected using the three variable selection techniques to remove wavelengths in similar regions.

**Figure 10:**
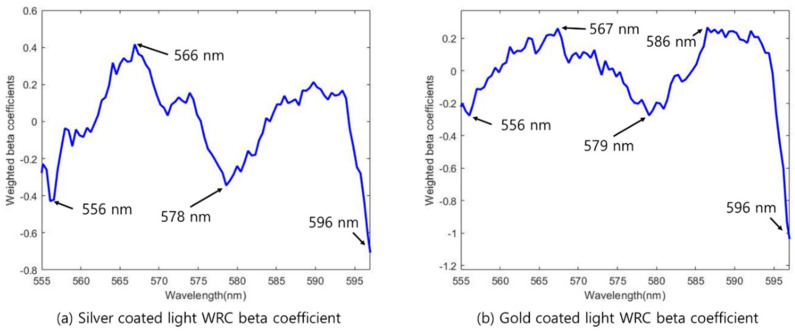
Selected bands using the WRC method: (**a**) silver−coated light WRC; (**b**) gold−coated light WRC.

First, through the WRC method, four and five bands were selected from the silver-coated and gold-coated models, respectively. For the chosen bands through the WRC method, wavelengths of 556, 566, 578, and 596 nm were selected in the silver-coated lamp model, and wavelengths of 556, 567, 579, 586, and 596 nm were selected in the gold-coated lamp model (Figure 10). The model using different lamps selected a similar wavelength region, with a difference of ±2 nm. Among them, the wavelengths of 578 nm and 579 nm extracted from the silver- and gold-coated lamp models were related to the effect of hemoglobin. A sharp difference in intensity was found between the wavelength of the yolk-destroyed eggs in the 596 nm region and that of normal eggs in the 600 nm region. This is due to the intensity difference between the different groups of samples. In the case of the gold-coated lamps, the beta coefficient in the 586 nm region was high, known as the PPIX wavelength.

For the second band selection method, sequential feature selection (SFS), the cutoff value was set to 3. In the silver-coated lamp data, 577, 597, and 598 nm were selected. Additionally, in the gold-coated lamp data, the same 577, 595, and 598 nm wavelengths were chosen. The wavelength of the corresponding region showed a similar trend, confirming that the acquired wavelength information corresponded to the hemoglobin region (Figure 10).

The silver-coated lamp model selected wavelengths of 577, 589, and 598 nm when using SPA, the third band selection method. In addition, for the gold-coated lamp model, wavelengths of 576, 593, and 598 nm were selected. The three-wavelength selection algorithms selected 9 and 10 wavelengths, including overlapping wavelengths in the silver coating and gold coating models, respectively. The wavelength information selected using the three-wavelength selection method is presented in Table 2. Wavelengths of 556, 566, 576, 577, 578, 589, 596, 597, and 598 nm were selected for the silver-coated lamp data, and wavelengths of 556, 567, 576, 577, 579, 586, 593, 595, 596, and 598 nm were selected for the gold-coated lamp data.

**Figure 11:**
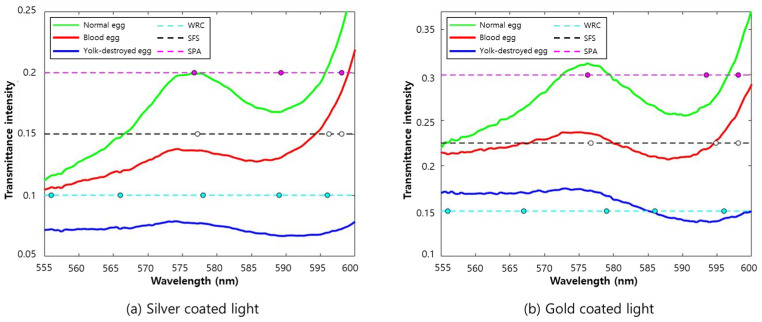
Graphical representation of the selected wavebands using the three variable selection methods. (**a**) Silver-coated halogen model; (**b**) gold-coated halogen model.

Although two different lamps were used, the wavelength ranges used to detect blood and abnormal eggs were similar. In addition, some regions overlap in the selected wavelength information. For example, the wavelengths of 576, 577, and 578 nm of the silver-coated lamp are in a nearly similar range, so there is a need for additional wavelength selection (Figure 11). In this study, a SR model was applied to the extracted wavelengths and analyzed for additional wavelength selection. *p* < 0.05, *p* < 0.01, *p* < 0.001, and *p* < 0.0001 were applied to the selected silver- and gold-coated lamp spectra, respectively, as threshold values. Six, five, four, and three wavelengths were selected for each threshold on the silver-coated lamp model, and seven, six, five, and three wavelengths were selected for the gold-coated lamp model (Table 3). Finally, the selected wavelength was used to build the final classification model.

#### 3.3.4. Model Performance

This study developed a model to evaluate previously selected wavebands. Construction of the PLS-DA model. Table 4 presents the results of the final PLS-DA model developed using the selected wavelengths. For the silver-coated lamp, the model classified normal and yolk-destroyed eggs with 100% accuracy. However, in the case of bloody eggs, the classification accuracy decreased from 92.6% to 82% as the number of wavelengths decreased. The total accuracy decreased from 97.5% to 94% as the accuracy of bloody egg classification decreased. The four wavelengths finally selected in the silver-coated lamp model were 577, 589, 596, and 598 nm, and 577 nm was the wavelength range associated with hemoglobin. In addition, in the case of 589 nm, as the wavelength range of PPIX, the corresponding wavelength range was also confirmed to affect model construction. However, the 589 nm waveband was removed by lowering the stepwise threshold to 0.0001. In addition, the wavelength region of 596 and 598 nm is an area in which the wavelength intensities of normal eggs and yolk-destroyed eggs are significantly different, and the band is also considered to be a wavelength region that helps detect internal abnormalities with characteristics of decreasing light transmittance.

It was confirmed that the accuracy of the overall gold-coated lamp model was inferior to that of the silver-coated lamp model. In addition, for the gold-coated lamp, five wavelength regions (556, 576, 593, 595, and 598 nm) were considered important when the same threshold of 0.001 was applied. By lowering the threshold of SR to 0.0001, three major wavebands (577, 595, and 598 nm) were selected. In the case of the gold-coated lamp, unlike the silver-coated lamp model, the accuracy tended to be relatively low, in the range of 95.7–98.6% when detecting yolk-destroyed eggs. When classifying bloody eggs, the accuracy gradually decreased as the number of bands decreased (78–89%), and it was confirmed that the accuracy of bloody egg classification affects the overall accuracy. Based on the overall classification accuracy, silver coating is useful for reducing to six wavelengths with an accuracy of 95% or more. When constructing an economical device, this method has more advantages than a gold-coated lamp because it requires less than 10 wavebands.

## 4. Conclusions

This study optimized (1) the light source, (2) the angle, and (3) wavebands for distinguishing abnormal eggs from normal ones. This study constructed classification models using gold- and silver-coated halogen lamps in stationary devices. With the wavelength selection method, it was possible to classify abnormal eggs (bloody eggs and yolk-destroyed eggs) with an overall accuracy of 93.7–95.9% with a silver-coated halogen lamp and with an accuracy of 91.1–95.9% with a gold-coated halogen lamp. It was found that, when evaluating on the basis of model accuracy, the appropriate number of bands in the silver-coated lamp-based model is 6 wavelengths and the appropriate number of bands in the gold-coated model is 10. The results can be used to construct an economic spectrometer that handles a particular wavelength range instead of the entire spectral range.

Our research findings show the possibility of replacing the halogen-tungsten light sources with LEDs. This is because LED utilization simplifies the overall system and reduces computing time by reducing unnecessary signal information. We anticipate that the results of this study will be used as primary research for optimizing abnormal egg detection systems. Although this study showed good performance for optimizing the system parameters in a pilot study, it still needs to confirm the system parameters and the developed model to be used in the condition of high-speed sample transition of the industry sorting environment, which can be the future work of this study.

## Figures and Tables

**Table 1:** The whole spectrum range PLS-DA model using the different light sources (gold and silver) for abnormal eggs classification.

Total Accuracy (%)										
Experiment	Light Source	Pre-Processing Method	Calibration Set				Validation Set			
			Normal	Bloody	YB	Total	Normal	Bloody	YB	Total
Condition 1	Gold	Raw	95.1	85.1	81.7	87.3	92.1	85.4	78.1	85.2
		Mean norm	81.4	86.5	76.4	81.4	76.3	91.3	66.7	78.4
		SG 1st	94.1	82.0	85.6	87.2	89.5	75.7	77.1	80.8
	Silver	Raw	94.4	96.3	100.0	96.9	100.0	92.7	97.8	96.8
		Mean norm	100.0	92.7	97.8	96.8	100.0	92.7	93.5	95.4
		Max norm	100.0	92.7	93.5	95.4	94.4	90.2	97.8	94.2
Condition 2	Gold	Raw	88.9	94.0	100.0	94.3	84.0	96.4	100.0	93.5
		Mean norm	100.0	94.5	100.0	98.2	98.7	97.3	100.0	98.7
		Max norm	100.0	95.9	100.0	98.6	96.0	96.4	100.0	97.5
	Silver	Raw	87.8	84.5	100.0	90.8	89.2	87.0	100.0	92.0
		Mean norm	93.9	86.0	99.4	93.1	97.3	90.2	100.0	95.8
		Range norm	96.9	88.9	100.0	95.3	97.4	92.3	100.0	96.6
Condition 3	Gold	Raw	91.2	94.8	100.0	95.3	88.6	89.7	98.7	92.3
		Mean norm	100.0	96.2	98.3	98.2	100.0	93.1	97.3	96.8
		MSC	100.0	95.3	96.0	97.7	100.0	94.3	96.0	96.8
	Silver	Raw	89.4	90.7	99.3	93.1	95.1	89.3	95.9	93.4
		Mean norm	88.3	88.0	100.0	92.1	90.2	88.1	98.6	92.3
		Range norm	90.4	90.3	100.0	93.6	92.7	85.7	100.0	92.8

Condition 1: the sensor is on the top of the egg’s blunt end, and the light comes in from a position perpendicular to the sensor; condition 2: the sensor is on top of the blunt end of the egg, and the light-emitting part illuminates the egg at an angle of 45 degrees; condition 3: the sensor is tilted 45 degrees away from the blunt end of the egg, and the light is illuminated from the side of the egg (Figure 4a); Raw: raw spectrum; Mean norm: Mean normalization; Max norm: Max normalization; Range norm: Range normalization; SG 1st: Savitzky-Golay 1st derivative; MSC: Multiplicative Scatter Correction.

**Table 2:** The selected wavelengths for identifying the abnormal eggs using different variable selection methods.

Lamp Type	Variable Selection Method	Selected Variable Numbers	Selected Wavelengths (nm)
Silver Lamp	WRC	4	556. 566, 578, 596
	SFS	3	577, 597, 598
	SPA	3	577, 589, 598
	Total band	9	556, 566, 576, 577, 578, 589, 596, 597, 598
Gold Lamp	WRC	5	556, 567, 579, 586, 596
	SFS	3	577, 595, 598
	SPA	3	576, 593, 598
	Total	10	556, 567, 576, 577, 579, 586, 593, 595, 596, 598

**Table 3:** Band selection results using a forward stepwise regression model to a gold- and silver-coated lamp model.

Lamp Type	Stepwise P Value Threshold	Selected Variable Numbers	Selected Wavelengths (nm)
Silver Lamp	0	9	556, 566, 576, 577, 578, 589, 596, 597, 598
	0.05	6	556, 566, 577, 589, 596, 598
	0.01	5	556, 577, 589, 596, 598
	0.001	4	577, 589, 596, 598
	0.0001	3	577, 596, 598
Gold Lamp	0	10	556, 567, 576, 577, 579, 586, 593, 595, 596, 598
	0.05	7	556, 576, 577, 586, 593, 595, 598
	0.01	6	556, 576, 577, 593, 595, 598
	0.001	5	556, 577, 593, 595, 598
	0.0001	3	577, 595, 598

**Table 4:** Selected bands of the PLS-DA model results using silver and gold tungsten lamps for the discrimination of abnormal eggs.

Lamp Type	Selected Bands	Validation Set Accuracy			
		Normal (%)	Bloody (%)	YD (%)	Total (%)
Silver Lamp	9	100.0	92.6	100.0	97.5
	6	100.0	86.6	100.0	95.5
	5	100.0	83.8	100.0	94.6
	4	100.0	82.0	100.0	94.0
	3	100.0	82.5	98.7	93.7
Gold Lamp	10	100.0	89.0	98.6	95.9
	7	100.0	82.3	95.7	92.7
	6	100.0	79.8	98.2	92.7
	5	100.0	78.3	98.4	92.2
	3	100.0	78.0	95.3	91.1

Normal (%): normal egg prediction accuracy (%); Bloody (%): bloody egg prediction accuracy (%); YD (%): yolk-destroyed egg prediction accuracy (%); Total (%): total egg group prediction accuracy (%).

## Data Availability

Data sharing is not applicable to this article.
